# On Tracheotomy in Croup, and the Measures Adapted to Secure Success from This Operation

**Published:** 1857-01

**Authors:** M. A. Trousseau

**Affiliations:** Professor of Clinical Medicine, Faculty of Medicine, Paris, etc.


ART. VIII.— *On Tracheotomy in Croup, and the Measures adapted to secure success from this operation.* By M. A. Trousseau, Professor of Clinical Medicine, Faculty of Medicine, Paris, etc. (From *Annuaire des Sciences Médicales*, par M. M. Lorain et Robin, première année. 1856.)

It is well known, that the operation of tracheotomy in croup was first successfully practiced by M. Brettenneau. The operation was imported into Paris, by M. Trousseau, when only a single cure could be cited in its favor. Received with distrust by the surgeons, and by some of them rejected, failing in the hands of several, this heoric method has now triumphed over opposition. M. Trousseau has labored for its success, receiving it from his master and making it his own, and now that its opponents are silent, and every one is on his side, he does not think that he has accomplished enough, but his ardor in its behalf is in no wise diminished. "When," he says, "in the course of a medical life already long, any one has contributed his part in the diffusion of an useful idea, if in each year numerous facts come to be added to other facts of the same kind, and if the success attained by a therapeutical measure is constantly increasing, the result is, that he becomes obstinate in his apostleship and devotes himself, even in old age, to the cause which he has espoused."

It is in France that tracheotomy has the most active partizans. England is behind us in this respect. It is sufficient to quote the figures cited by M. Trousseau, to be convinced that tracheotomy practiced in the advanced stage of croup, is frequently followed by success.

During the year 1854, M. Trousseau operated nine times. Two infants died; seven were cured, and are now living and well.

"It is true," remarks M. Trousseau, "the proportion, of course, is not always as large; but if I aggregate the operations which I have performed during the last four years, I find the number to be twenty-four, and the number of cures fourteen: that is to say, more than one-half. At the Hospital for Sick Children, during the last five years, the proportion of cures has been nearly one-quarter. The following are the figures contained in the official returns from this hospital for the last five years: In 1850, twenty operations and six cures, nearly a third. In 1851, thirty-one operations and twelve cures, more than a third. In 1852, fifty-nine operations and eleven cures, a little less than a fifth. In 1853, sixty-four operations and seven cures. only a ninth. In 1854, forty-four cases and eleven cures, a quarter."

If it be considered that these operations were performed in a hospital, in an insalubrious atmosphere, and under unfavorable hygienic conditions, it cannot be doubted that a series of operations performed in private practice, either in the city or country, would give results much more satisfactory. M. Trousseau does not doubt that in private practice a proportion of one-half of cures will be found to be the rule, provided the operation be performed under circumstances in which a cure is possible. "If the diphtheritic infection has affected profoundly the organism; if the skin, and especially the nasal passages are the seat of a special phlegmesia; if the frequency of the pulse, the delirium, denote a great degree of poisoning; if the danger is in this general condition rather than in the local affection of the larynx or trachea, the operation should never be resorted to: it is invariably followed by death. On the other hand, when the local affection constitutes the chief source of danger, to whatever extent the asphyxia has attained, even if the child be within a few moments of death, tracheotomy succeeds nearly as well as if it had been resorted to three or four hours sooner."

M. Trousseau lays great stress on the manual operation of tracheotomy. It is not a question of an operation impracticable for physicians and reserved alone for the surgeons. Manual dexterity holds a small place as regards the results; but it is necessary to select the method which is least obnoxious to accidents such as hæmorrhage. The incisions should be made slowly, layer by layer being divided, turning aside the vessels and the muscles with a blunt hook, and exposing fully the trachea before opening it. The trachea should alone be opened, the larynx not being touched. The instruments indispensable for the operation, are an ordinary bistoury, hooks, a dilater and the double canula.

M. Trousseau is emphatic against too rapid performance of the operation. He is opposed to a celerity more brilliant than safe, which exposes the patient to the occurrence of terrible hæmorrhage. "Pains should be taken to place between the skin and the canula introduced, a shield of waxed cloth or of caoutchouc, in order to prevent the edges of the canula and the ribbons which are attached to it, from irritating the wound. The internal canula should be taken out and replaced, after having been cleansed, every two or three hours. The neck of the child should be enveloped with a knitted woollen muffler, or a large piece of muslin, in order that the air inspired may be warm and impregnated with the warm vapor furnished by the expired air. This is an important point. Moreover, in this way is avoided dryness of the canula and of the trachea."

In addition to these points, the wound should be cauterized with the nitrate of silver, in order to prevent the diphtheritis which may be developed there.

The point with which M. Trousseau is chiefly pre-occupied in the management after the operation relates to hygiene. The patients must be well nourished; blisters and bleedings are to be prescribed and the forces are to be sustained. Some patients experience great difficulty in deglutition, liquids getting access into the glottis. It is, therefore, preferable to give aliment in a semi-solid form, such as soups made very thick. *

